# Cannabinoid Regulation of Acute and Anticipatory Nausea

**DOI:** 10.1089/can.2016.0006

**Published:** 2016-04-01

**Authors:** Erin M. Rock, Martin A. Sticht, Cheryl L. Limebeer, Linda A. Parker

**Affiliations:** ^1^ Department of Psychology and Collaborative Neuroscience Graduate Program, University of Guelph, Guelph, Canada.; ^2^ Department of Physiology and Pharmacology, Hotchkiss Brain Institute, University of Calgary, Calgary, Canada.

**Keywords:** 2-arachidonoylglycerol, acute nausea, anandamide, anticipatory nausea, CB_1_ receptor, conditioned gaping, endocannabinoid

## Abstract

Chemotherapy-induced nausea is one of the most distressing symptoms reported by patients undergoing treatment, and even with the introduction of newer antiemetics such as ondansetron and aprepitant, nausea remains problematic in the clinic. Indeed, when acute nausea is not properly managed, the cues of the clinic can become associated with this distressing symptom resulting in anticipatory nausea for which no effective treatments are available. Clinical trials exploring the potential of exogenous or endogenous cannabinoids to reduce chemotherapy-induced nausea are sparse; therefore, we must rely on the data from pre-clinical rat models of nausea. In this review, we explore the human and pre-clinical animal literature examining the potential for exogenous and endogenous cannabinoid treatments to regulate chemotherapy-induced nausea. The pre-clinical evidence points to a compelling need to evaluate the antinausea potential of cannabidiol, cannabidiolic acid, and treatments that boost the functioning of the endocannabinoid system in human clinical trials.

## Introduction

For more than 5000 years, cannabis has been utilized as a medicine (see Ref.^1^), including for the treatment of nausea and vomiting. In response to their inability to manage patients' chemotherapy-induced nausea and vomiting with conventional antiemetics, oncologists began to evaluate the antiemetic properties of cannabis in the late 1970s, following anecdotal reports of smoked cannabis alleviating chemotherapy-induced nausea and vomiting. In addition, the synthetic cannabinoid agonists, nabilone (Cesamet^®^) and dronabinol (Marinol^®^), were subsequently evaluated and approved for their antiemetic and antinausea properties in chemotherapy patients.^2^

Currently, vomiting is relatively well managed in the clinic since the advent of the 5-hydroxytryptamine 3 (5-HT_3_) receptor antagonists (such as ondansetron) and the neurokinin-1 (NK-1) receptor antagonists (such as aprepitant)^3^; however, nausea and anticipatory nausea (a conditioned response through which simply returning to the treatment clinic causes patients to feel nauseous as a result of their association between the contextual cues of the clinic and the nausea they experience from treatment) are still not properly managed.^3^ Nausea remains as one of the most distressing symptoms experienced by cancer patients undergoing chemotherapy treatment,^4^ highlighting the need for alternative pharmacotherapies to be explored.

Pre-clinical animal models of nausea are necessary to evaluate putative antinausea compounds. One such selective and reliable rodent model is nausea-induced conditioned gaping. Although rodents are incapable of vomiting, they display conditioned gaping reactions in response to a flavor previously paired with an illness-inducing agent such as lithium chloride (LiCl).^5^ They also avoid drinking this flavor as a measure of taste avoidance. However, conditioned gaping reactions are indicative of nausea in rodents, because, unlike taste avoidance, only emetic drugs produce conditioned gaping in rats, and antiemetic treatments (including cannabinoids) block conditioned gaping.^6^ Rats avoid drinking a flavor paired even with a rewarding drug.^6^

## Cannabinoids in Human Patients

### Exogenous cannabinoids and chemotherapy-induced acute nausea

Delta-9-tetrahydrocannabinol (THC), the major psychoactive component of cannabis,^7,8^ is a high-affinity agonist for both the cannabinoid 1 (CB_1_) and cannabinoid 2 (CB_2_) receptors and it has been shown to be effective in reducing chemotherapy-induced vomiting^9^ and/or nausea^10–20^ when smoked or orally administered.

Dronabinol (Marinol), an orally administered synthetic THC, has been shown to be effective in reducing chemotherapy-induced nausea and/or vomiting.^21–23^ In 1985, nabilone (Cesamet), another orally administered synthetic THC, was approved for nausea and vomiting only in patients who were unresponsive to conventional treatments. Nabilone has also been shown to reduce chemotherapy-induced nausea and/or vomiting.^24–38^ Please refer to Table 1 for more specific details of these findings. These findings highlight the potential of CB_1_ receptor agonism to reduce chemotherapy-induced nausea and/or vomiting, over that of classic antiemetic treatments.

**Table 1.** **Efficacy of Various Exogenous Cannabinoids to Alleviate Acute Nausea in Humans**

Compound	References	Efficacy	Dose	Nausea-evoking agent	Sample details
THC	Chang et al.^10^	Compared to placebo:• More effective	Smoked THC (1.93% THC ∼17.4 mg), oral THC (10 mg/m^2^)	Methotrexate (250 mg/kg)	15 patients (10 males, 5 females; 15–49 years old)
	Ekert et al.^11^	Compared to D_2_ receptor antagonists:• More effective	Oral THC (10 mg/m^2^)	Various chemotherapy agents	33 patients (22 males, 11 females; 5–19 years old)
	Frytak et al.^12^	Compared to placebo:• More effective	Oral THC (15 mg)	Various chemotherapy agents	116 patients (70 males, 46 females; 21–70+ years old)
		Compared to D_2_ receptor antagonist:			
		• As effective			
	Kluin-Neleman et al.^13^	Compared to placebo:• More effective	Oral THC (10 mg/m^2^),	Chlormethine (6 mg/m^2^) and vincristine (1.4 mg/m^2^) with procarbazine (100 mg/m^2^) and prednisone (40 mg/m^2^)	11 patients (10 males, 1 female; 21–53 years old)
	Lucas and Laszlo^14^	Compared to D_2_ receptor antagonist:• More effective	Oral THC (15 mg/m^2^, or 4 mg/m^2^)	Details not provided	53 patients
	McCabe et al.^15^	Compared to D_2_ receptor antagonist:• More effective	Oral THC (15 mg/m^2^)	Various chemotherapy agents	36 patients (9 males, 27 females; 18–69 years old) refractive to antiemetics
	Neidhart et al.^16^	Compared to D_2_ receptor antagonist:	Oral THC (10 mg)	Cisplatinum, nitrogen mustard, or doxorubicin	73 patients (42 males, 31 females
		• As effective			
	Orr et al.^17^	Compared to placebo:• More effective	Oral THC (7 mg/m^2^)	Various chemotherapy agents	55 patients (28 males, 51 females; 22–71 years old) refractive to antiemetics
		Compared to D_2_ receptor antagonist:• More effective			
	Orr and McKernan^18^	Compared to placebo:• More effective	Oral THC (7 mg/m^2^),	Various chemotherapy agents	79 patients (22–71 years old) refractive to anti-emetics
		Compared to D_2_ receptor antagonist:			
		• More effective			
Dronabinol (Marinol^®^)	Lane et al.^22^	Compared to D_2_ receptor antagonist:• As effective	Oral dronabinol (10 mg)	Various chemotherapy agents	62 patients (29 males, 33 females; (20–68 years old)
	Meiri et al.^23^	Compared to placebo:• More effective	Oral dronabinol (2.5, 5 mg)	Various chemotherapy agents	61 patients (24 males, 37 females; 24–81 years old)
		Compared to 5-HT_3_ receptor antagonist: • As effective			
Nabilone (Cesamet^®^)	Ahmedzai et al.^24^	Compared to D_2_ receptor antagonist:• More effective	Oral nabilone (2 mg)	Cyclophosphamide (1g/m^2^), adriamycin (40 mg/m^2^), and etoposide (100 mg/m^2^)	34 patients (19 males, 15 females; 27–72 years old)
	Dalzell et al.^26^	Compared to D_2_ receptor antagonist:	Oral nabilone (1–3 mg)	Various chemotherapy agents	18 patients (14 males, 4 females; 0–17 years old)
		• More effective			
	Einhorn et al.^27^	Compared to D_2_ receptor antagonist:	Oral nabilone (2 mg)	Various chemotherapy agents	80 patients (15–74 years old)
		• More effective			
	Herman et al.^30^	Compared to D_2_ receptor antagonist:	Oral nabilone (2 mg)	Various chemotherapy agents	152 patients (126 men, 26 women; 15–70 years old)
		• More effective			
	Johansson et al.^31^	Compared to D_2_ receptor antagonist:	Oral nabilone (2 mg)	Cisplatinum (50 mg/m^2^), adriamycin (40 mg/m^2^), cyclophosphamide (500 mg/m^2^)	26 patients (18–70 years old) refractive to antiemetics
		• More effective			
	Jones et al.^32^	Compared to placebo:	Oral nabilone (2 mg)	Various chemotherapy agents	54 patients (35 men, 19 women; 38–57 years old)
		• More effective			
	Levitt^28^	Compared to placebo:• More effective	Oral nabilone (2 mg)	Various chemotherapy agents	36 patients (12 men, 24 women; 17–78 years old)
	Niederle et al.^34^	Compared to D_2_ receptor antagonist:	Oral nabilone (2 mg)	Cisplatin (20 mg/m^2^) and adriamycin (600 mg/m^2^)	20 patients (male; 19–45)
		• More effective			
	Niiranen and Mattson^35^	Compared to D_2_ receptor antagonist:	Oral nabilone (1 mg)	Various chemotherapy agents	24 patients (20 males, 4 females; 48–78 years old)
		• As effective			
	Pomeroy et al.^36^	Compared to D_2_ receptor antagonist:	Oral nabilone (1 mg)	Various chemotherapy agents	38 patients (23 males, 15 females; 21–66 years old)
		• As effective			
	Steele et al.^37^	Compared to D_2_ receptor antagonist:	Oral nabilone (2 mg)	Various chemotherapy agents	37 patients (19–65 years old)
		• More effective			
	Wada et al.^38^	Compared to placebo:	Oral nabilone (2 mg)	Various chemotherapy agents	114 patients (47 males, 67 females; 18–81 years old)
		• More effective			
Sativex^®^	Duran et al.^39^	Compared to standard antiemetic treatment (corticosteroid/5-HT_3_ receptor antagonist or D_2_ receptor antagonist:	Oromucosal spray (2.7 mg THC +2.5 mg CBD/spray)		16 patients (1 male, 15 females; 34–76 years old) refractive to antiemetics
		• As effective			

CBD, cannabidiol; 5-HT_3_, 5-hydroxytryptamine 3; THC, delta-9-tetrahydrocannabinol.

Most recently, the oromucosal cannabis-based medicine, Sativex^®^ (1:1, THC:cannabidiol [CBD]), when combined with the standard treatment of a 5-HT_3_ antagonist and a corticosteroid, reduced delayed nausea (and vomiting).^39^ Because Sativex contains both THC and CBD, it is unknown which compound (or both) contributed to its antinausea effects. Moreover, recent findings in our laboratory indicate that subthreshold doses of THC and cannabidiolic acid (CBDA), the acidic precursor of CBD, when combined, effectively reduce acute nausea and anticipatory nausea in rats^40^; however, we have not investigated whether these effects are mediated by the action of THC at the CB_1_ receptor, CBDA at the 5-HT_1A_ receptor,^41^ or both.

These findings highlight the therapeutic potential of exogenously administered cannabinoids such as THC to reduce chemotherapy-induced nausea. It is important to note here, the unique ability of cannabinoids, to effectively manage nausea, a symptom that current antiemetic treatments cannot control.

### Endocannabinoid levels during the experience of nausea in humans

To date, there have been no published clinical trials investigating whether endocannabinoid manipulations (such as increased action of anandamide [AEA] and 2-arachidonylglyercol [2-AG] through enzyme inhibition of fatty acid amide hydrolase [FAAH] or monoacylglycerol lipase [MAGL]) reduce nausea; however, changes in endocannabinoid levels have been measured due to nausea-inducing manipulations. For example, decreases in AEA levels have been reported with administration of the anesthesia sevoflurane, which results in postoperative nausea.^42^ In addition, reduced levels of AEA and 2-AG have been shown in those experiencing motion sickness.^43^ Therefore, it seems that endogenous cannabinoids may be important neuromodulators involved in the experience of nausea, with decreased levels of AEA and/or 2-AG evident with nausea-inducing manipulations. Further research needs to clarify how the endogenous cannabinoid system is involved in the experience of nausea, and more specifically, how manipulations of this system could attenuate chemotherapy-induced nausea.

### Exogenous cannabinoids and chemotherapy-induced anticipatory nausea

Anticipatory nausea is a conditional association between the chemotherapy clinic cues and the nausea-inducing chemotherapeutic treatment such that patients experience nausea upon returning to the clinic where illness-inducing treatment was administered.^44^ Anticipatory nausea develops in 25–59% of chemotherapy patients,^44–51^ if acute nausea has not been properly managed. Once established, anticipatory nausea is refractive to treatment with the classic 5-HT_3_ receptor antagonists such as ondansetron,^4,52–54^ and patients are currently prescribed sedating antianxiety drugs (benzodiazepines).^55,56^ Clearly, there is a great need for alternative therapeutics for anticipatory nausea as current medicines are insufficient.

In the only published clinical trial to date assessing cannabinoids and anticipatory nausea, Lane et al.^22^ showed that dronabinol was ineffective in reducing anticipatory nausea, but it is important to note that 86% of the patients included in the study were being given highly emetogenic chemotherapeutic treatments. Although dronabinol may not be as effective for anticipatory nausea resulting from highly emetogenic agents, it may be effective in less emetogenic chemotherapy regimens.

As proper management of acute nausea is the best prevention for the development of anticipatory nausea, the efficacy of THC and its synthetic derivatives in reducing acute nausea (as discussed in the section “Exogenous cannabinoids and chemotherapy-induced acute nausea”) should reduce the risk of anticipatory nausea developing. Clinical trials are necessary to evaluate THC, as well as other phytocannabinoids such as CBD, for their ability to reduce acute and/or anticipatory nausea, especially in comparison to the current first-line treatment (5-HT_3_ receptor antagonist/dexamethasone/NK-1 receptor antagonist).

### Endogenous cannabinoids and chemotherapy-induced anticipatory nausea

Cannabinoid compounds are effective in reducing acute nausea in human patients (as discussed in the section “Exogenous cannabinoids and chemotherapy-induced acute nausea”) and anticipatory nausea in animal models (as discussed in the section “Exogenous cannabinoids reduce anticipatory nausea in rats”), but no published clinical trials have evaluated enzyme inhibitors in anticipatory nausea patients. Such investigations have relied solely on animal models, highlighting the need for clinical trials.

## Cannabinoids in Animal Models of Nausea

Considerable evidence implicates the endocannabinoid system in the regulation of nausea in the animal model of conditioned gaping reactions in rats.^57^ Here, we review the experimental pre-clinical evidence for the potential of cannabinoids and manipulation of the endocannabinoid system to reduce both acute^57^ and anticipatory nausea^58^ based upon the conditioned gaping models. Please refer to Table 2 for more specific details of these findings.

**Table 2.** **Efficacy of Various Exogenous and Endogenous Cannabinoids to Alleviate Nausea-Induced Conditioned Gaping and Contextually Elicited Conditioned Gaping in Rats**

Compound	Dose details	Efficacy in acute nausea-induced gaping	Efficacy in contextually elicited gaping
CB_1_ receptor agonists			
THC	0.5 mg/kg, i.p. 30 min pretreatment	Compared to VEH: • More effective (Limebeer and Parker^59^; Parker and Mechoulam^60^; Parker et al.^61^)	Compared to VEH: • More effective (Limebeer et al.^78^; Rock et al.^79^)Compared to 5-HT_3_ receptor antagonist: • More effective (Rock et al.^79^)
Endocannabinoid manipulations			
Anandamide		Not evaluated	Not evaluated
2-AG	1.5, 2 mg/kg, i.p. 15 min pretreatment 0.5, 1 μg, bilaterally, after acute nausea test	Compared to VEH: • More effective (Sticht et al.^67^) Administration to the IC, compared to VEH: • More effective (Sticht et al.^57^)	Not evaluated
FAAH inhibition			
PF-3845	10 mg/kg, i.p. 120 min pretreatment 2 ug, bilaterally, 30 or 70 min pretreatment	Compared to VEH: • More effective (Rock et al.^64^) Administration to the IC, compared to VEH: • As effective (Sticht et al.^76^)	Compared to VEH: • More effective (Rock et al.^64^) Administration to the IC, compared to VEH, 5-HT_3_ receptor antagonist: • As effective (Limebeer et al.^83^)
MAGL inhibition			
MJN110	10, 20 mg/kg, i.p. 120 min pretreatment 2 ug, bilaterally, 30 or 70 min pretreatment	Compared to VEH: • More effective (Parker et al.^68^) Administration to the IC, compared to VEH: • More effective (Sticht et al.^76^)	Compared to VEH: • More effective (Parker et al.^68^) Administration to the IC, compared to VEH: 5-HT_3_ receptor antagonist: • More effective (Limebeer et al.^83^)
Dual FAAH/MAGL inhibition			
JZL195	10 mg/kg, i.p. 120 min pretreatment	Not evaluated	Compared to VEH: • More effective (Limebeer et al.^82^)

CB_1_, cannabinoid 1; 2-AG, 2-arachidonylglyercol; FAAH, fatty acid amide hydrolase; IC, insular cortex; MAGL, monoacylglycerol lipase.

### Exogenous cannabinoids reduce acute nausea-induced conditioned gaping

THC attenuates the establishment of acute nausea-induced conditioned gaping induced by the chemotherapy drug cyclophosphamide,^59^ as well as with LiCl,^60,61^ through a CB_1_ receptor-mediated effect. Thus, as demonstrated in humans, THC (through CB_1_ receptor agonism) has an antinausea effect in the rat conditioned gaping model (acute nausea).

It is interesting to note that two nonpsychoactive cannabinoids found in cannabis, CBD^60^ and its precursor CBDA,^41^ also interfere with acute nausea-induced conditioned gaping in rats without impairing the locomotor activity. CBDA was 1000 times more potent than CBD in reducing acute nausea.^62^ Unlike THC, however, the antinausea effect of CBD^63^ and CBDA^41^ was mediated by agonism of 5-HT_1A_ receptors, not CB_1_ receptors. Furthermore, subthreshold doses of CBDA potentiated the antinausea effect of the 5-HT_3_ receptor antagonist, ondansetron.^62^ These findings suggest that CBDA, in particular, may be a highly effective treatment for acute nausea alone or in combination with conventional treatments, although it has not yet been evaluated in clinical trials.

### Endogenous cannabinoids reduce acute nausea-induced conditioned gaping

Recent studies in our laboratory have investigated the role of the endogenous cannabinoid system in acute nausea-induced conditioned gaping, utilizing enzyme inhibitors that increase AEA and 2-AG levels (through inhibition of FAAH or MAGL, respectively). PF-3845, a novel FAAH inhibitor, reduces acute nausea-induced conditioned gaping; however, this effect was reversed by a peroxisome proliferator-activated receptor alpha (PPARα) receptor antagonist, not a CB_1_ receptor antagonist.^64^ It is likely that this antinausea effect is due to increases in oleoylethanolamide (OEA) and palmitoylethanolamide (PEA) following PF-3845 administration.^65^ Further investigation of the effect of fatty acids other than AEA on acute nausea is thus warranted. However, AEA may also be involved in the antinausea effect of FAAH inhibition, because the FAAH inhibitor, URB597, potentiated the antinausea effect of systemic AEA administration and this effect was reversed by CB_1_ receptor antagonism.^66^

Exogenous 2-AG administration (which is rapidly deactivated by MAGL) reduces acute nausea-induced conditioned gaping.^67^ MJN110, a MAGL inhibitor, also reduces acute nausea-induced conditioned gaping, a CB_1_ receptor-mediated effect.^68^ The aforementioned results, pertaining to systemic administration of enzyme inhibitors, suggest a role of the endogenous cannabinoid system in the suppression of nausea, but the specific brain region(s) critical for nausea are still not completely clear.

A brain region of interest for nausea is the interoceptive insular cortex (IC), an area shown to be involved in nausea,^69^ as stimulation of the IC^70–72^ and functional neuroimaging studies in humans^73,74^ pinpoint the IC as a critical region for nausea.

Our laboratory has begun to investigate how the endogenous cannabinoid system mediates nausea, with a specific focus on the rat interoceptive IC. Indeed, administration of the synthetic cannabinoid, HU-210, into the interoceptive IC reduces conditioned gaping through a CB_1_ receptor-mediated effect.^75^ Furthermore, administration of 2-AG to the interoceptive IC reduces conditioned gaping,^57^ and administration of the MAGL inhibitor MJN110 into the interoceptive IC (but not the FAAH inhibitors URB597 or PF-3845) reduces conditioned gaping, a CB_1_ receptor-mediated effect.^76^ These results suggest that the effects of the endocannabinoid system during an experience of acute nausea may be mediated by 2-AG (and not AEA) in the interoceptive IC.

### Exogenous cannabinoids reduce anticipatory nausea in rats

In addition to displaying conditioned gaping to a nausea-paired flavor, rats also display conditioned gaping when returned to a nausea-paired context; a phenomenon analogous to human anticipatory nausea.^77^ Furthermore, much like with human anticipatory nausea, ondansetron does not reduce contextually elicited conditioned gaping in rats.^78,79^ Also, similar to human anticipatory nausea, administration of benzodiazepine does reduce contextually elicited conditioned gaping in rats, but also impairs locomotor activity.^79^ In contrast, low doses of THC reduce contextually elicited gaping in the absence of impaired locomotion,^78,79^ indicating that THC may be a superior therapeutic, over sedating benzodiazepines, in treating anticipatory nausea.

As with acute nausea, both CBD^80^ and CBDA^41,79^ reduce anticipatory nausea in this pre-clinical model by a 5-HT_1A_ receptor mechanism of action, with CBDA about 1000 times more potent than CBD.^79^ Neither CBD nor CBDA interfered with motor activity. Given that these compounds are nonpsychoactive, future clinical trials with human patients are gravely needed as there are currently no specific treatments for anticipatory nausea in humans.

### Endogenous cannabinoids reduce anticipatory nausea in rats

The endogenous cannabinoid system has also been implicated in the control of anticipatory nausea (for review).^81^ The FAAH inhibitors URB597 or PF-3845 reduce contextually elicited conditioned gaping; unlike acute nausea, the antinausea effect of FAAH inhibition on anticipatory nausea was reversed by a CB_1_ receptor antagonist,^64,80^ presumably through AEA elevation. The MAGL inhibitor, MJN110, also reduces contextually elicited gaping in rats, a CB_1_ receptor-mediated effect.^68^ Finally, dual FAAH-MAGL inhibition with JZL195 reduces contextually elicited gaping by elevated AEA, PEA, and OEA,^82^ a CB_1_ receptor-mediated effect. Recent findings in our laboratory indicate that infusion of the MAGL inhibitor, MJN110 (but not the FAAH inhibitor PF-3845 nor ondansetron), into the interoceptive IC suppressed contextually elicited conditioned gaping, a CB_1_ receptor-mediated effect.^83^ These results suggest that the interoceptive IC may be a critical region for AN (in addition to acute nausea), mediated by 2-AG activity at the CB_1_ receptor.

## Conclusions

The endocannabinoid system clearly plays an important role in the regulation of nausea. The pre-clinical findings suggest that CB_1_ receptor agonists, as well as FAAH and MAGL inhibitors, which elevate levels of AEA and 2-AG, respectively, reduce acute nausea and anticipatory nausea. As well, by a noncannabinoid mechanism of action, both CBD and CBDA are highly effective antinausea treatments in these animal models without producing sedation or psychoactive effects. Nausea remains an elusive, difficult to control symptom in human chemotherapy patients and there are currently no selective treatments for anticipatory nausea. Clinical trials with FAAH inhibitors, MAGL inhibitors, CBD, and CBDA are warranted to improve the quality of life of patients undergoing cancer treatment by reducing the side effects of nausea and anticipatory nausea when it develops.

## Abbreviations Used

2-AG: 2-arachidonylglyercol
5-HT_3_: 5-hydroxytryptamine 3
AEA: anandamide
CB_1_: cannabinoid 1
CBD: cannabidiol
CBDA: cannabidiolic acid
FAAH: fatty acid amide hydrolase
IC: insular cortex
LiCl: lithium chloride
MAGL: monoacylglycerol lipase
NK-1: neurokinin-1
OEA: oleoylethanolamide
PEA: palmitoylethanolamide
THC: delta-9-tetrahydrocannabinol
